# Comparative Transcriptomics Suggests that Breast Secretory Epithelium Reuses Gene Repertoires Conserved Across Vertebrates Beyond Mammals

**DOI:** 10.1093/gbe/evag130

**Published:** 2026-05-29

**Authors:** Marie Saitou

**Affiliations:** Department of Animal and Aquacultural Sciences, Faculty of Biosciences, Norwegian University of Life Sciences (NMBU), Ås, Norway

**Keywords:** comparative transcriptomics, breast tissue, epithelial secretory tissues, organ evolution, vertebrate gene conservation, module preservation

## Abstract

The emergence of novel organs is often attributed to lineage-specific innovation; however, accumulating evidence suggests that many complex traits arise through the regulatory redeployment of conserved molecular components. From a molecular evolutionary perspective, an unresolved question is how preexisting gene repertoires are reorganized to generate tissue-specific functions. Here, we examine the molecular evolutionary organization of gene expression programs in human breast tissue by situating its transcriptome within a comparative framework of epithelial tissues with secretory functions. Using bulk RNA-seq data from the GTEx project, combined with co-expression network analysis, single-cell reference integration, and cross-vertebrate orthology assessment, we identify gene expression modules that are broadly shared across epithelial secretory tissues, relatively enriched in breast tissue, or biased toward female breast samples. We show that genes enriched in breast tissue are largely evolutionarily conserved across vertebrates and are embedded within transcriptional modules shared with other epithelial secretory organs. Co-expression analysis revealed partial overlap between breast modules and those of other epithelial secretory tissues, particularly in pathways related to secretion, vesicle trafficking, and extracellular matrix organization. Together, these results support a model in which breast secretory epithelium achieves tissue specialization through integration of conserved vertebrate gene repertoires, rather than through extensive emergence of mammal-specific genes.

Significance The mammary gland is a defining feature of mammals, but it remains unclear whether it emerged through entirely new genes or by reusing existing genes during evolution. We found that most genes active in human breast tissue are shared with other organs with secreting functions. These genes are observed across vertebrate species including nonmammalian species. These findings suggest that the mammary gland mainly evolved by reusing and reorganizing existing gene sets rather than relying on new, mammal-specific genes.

## Introduction

The emergence of novel organs is a central problem in evolutionary biology (Darwin and Wallace 1858; Mayr 1965; Dobzhansky 1982; Kimura 1985; Shubin et al. 2006; Wray 2007; Longo et al. 2013; Bi et al. 2021; Cupello et al. 2022). While phenotypic innovations are often described in terms of lineage-specific gene emergence or morphological transformation, growing evidence indicates that many complex traits arise through the regulatory reorganization and reuse of conserved molecular components (Ciliberti et al. 2007; Wagner 2011; Erwin 2021; Xia et al. 2025). However, how such regulatory reuse is structured at the transcriptome-wide level across related tissues remains understood.

The mammary gland, a hallmark trait of mammals, plays an essential role in mammalian offspring survival by providing nutrition and immunity through milk (Pond 1977; Peaker 2002; Hassiotou and Geddes 2013). The mammary gland is a secretory tissue that undergoes coordinated changes in growth, differentiation, and function from embryogenesis through adulthood and lactation (Medina 1996). Its development involves interactions among epithelial, stromal, and immune compartments, regulated by hormonal signaling and transcriptional control mechanisms (Dawson and Visvader 2021; Kaur et al. 2021; Rauner and Kuperwasser 2021; Slepicka et al. 2021). Extensive developmental and cellular studies have characterized mammary epithelial hierarchies, progenitor populations, and endocrine regulation associated with morphogenesis and milk production (Chen et al. 2017; Tharmapalan et al. 2019; Slepicka et al. 2021).

From an evolutionary perspective, the mammary gland represents a major organ-level innovation whose origin predates the diversification of extant mammalian lineages. Classical studies based on comparative morphology and histology, proposed that the mammary glands originated through the progressive modification of a single ancestral integumentary gland, typically hypothesized to be a sweat, sebaceous, or hair-associated gland (Bresslau and Hill 1920; Blackburn 1991; Moll et al. 2008; Picardo et al. 2009; Oftedal and Dhouailly 2013; Gegenbauer 2017). These models provided an important anatomical framework for understanding mammary gland origins.

In parallel, from the molecular evolution angle, comparative and phylogenetic studies have characterized a set of prominent milk-associated proteins and hormonal components across mammals, showing that broadly similar components are present across mammalian lineages (Enjapoori et al. 2014; Sharp et al. 2014; Kawasaki 2018). Such studies have been successful in disentangling the evolutionary histories of individual molecular components of lactation. However, these studies have not addressed how these components are transcriptionally organized into integrated gene expression programs that define mammary gland identity, nor how such programs relate to those of other epithelial tissues with secretory functions.

Anatomical and functional comparisons indicate that the mammary gland shares features with other tissues containing epithelial secretory components, pancreas, stomach, and skin (McManaman et al. 2006). These tissues differ substantially in physiological roles and regulatory organization (Khan et al. 2022), yet they provide an opportunity to examine how common and tissue-associated transcriptional programs are deployed across epithelial systems. Situating mammary gland gene expression within this broader landscape is essential for understanding whether mammary specialization reflects the emergence of novel gene repertoires or the differential deployment of ancestral regulatory modules (Brückner and Parker 2020).

Recent transcriptomic and single-cell studies have generated detailed descriptions of cellular composition and gene expression within individual tissues. In the mammary gland, single-cell RNA sequencing of tissue and milk has characterized epithelial and immune populations across developmental and physiological states (Li et al. 2020; Twigger et al. 2022). Comparable transcriptomic analyses have been performed for other secretory organs, including the salivary gland (Gao et al. 2018; Hauser et al. 2020; Saitou et al. 2020; Altrieth et al. 2024), pancreas (Ku et al. 2012; Bailey et al. 2016; Altrieth et al. 2024), stomach and intestinal glands (Elmentaite et al. 2021; Öling et al. 2024; Huang et al. 2025), and skin tissues (Inoue et al. 2022). However, these studies have largely addressed tissue-specific questions or descriptive cross-tissue comparisons, and relatively few analyses have examined how mammary gland gene expression relates to that of other epithelial secretory tissues within a unified comparative and evolutionary framework.

To address this gap, we examine transcriptome-wide gene expression patterns in human breast tissue in comparison with multiple epithelial tissues containing secretory components. By integrating bulk cross-tissue RNA-seq data with single-cell reference datasets, co-expression network analysis, and comparative genomic analyses, we investigate how tissue identity is assembled through the shared and differential deployment of gene expression programs. This approach enables us to assess whether mammary gland specialization is best understood as the regulatory reconfiguration of conserved epithelial modules, providing an evolutionary framework for interpreting organ-level novelty.

## Results

### Comparative Transcriptomic Analyses Reveal Patterns of Breast-tissue-associated Gene Expression

**Fig. 1.**
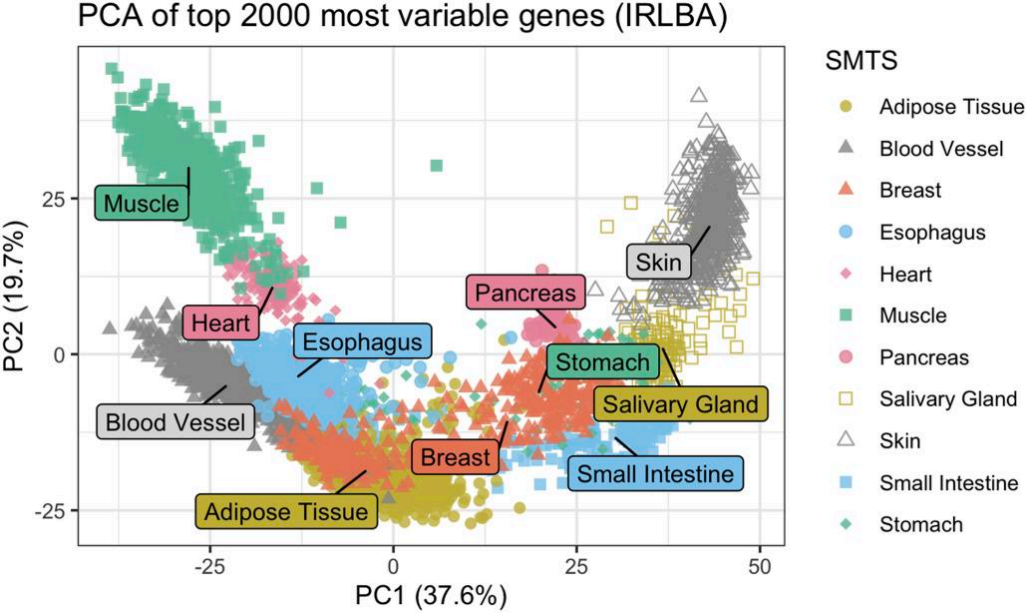
Principal component analysis (PCA) of tissue transcriptomes. PCA was performed using the 2,000 genes with the highest expression variance across tissues, to summarize global patterns of transcriptome variation across samples. Each point represents a tissue sample, and tissues are colored according to their anatomical source (legend, right). Tissues containing epithelial secretory components (breast, salivary gland, pancreas, stomach, small intestine, skin) cluster separately from nonglandular tissues (muscle, heart, blood vessel, esophagus, adipose). The *x* axis (PC1) and *y* axis (PC2) show the first and second principal components, which together explain the largest proportions of total expression variance (37.6% and 19.7%, respectively). Samples that are closer together in this plot have more similar global expression profiles, whereas those farther apart differ more strongly in their transcriptome composition. Colored clusters correspond to distinct tissue types. Labels indicate the centroids of major tissue groups.

Principal component analysis (PCA) was performed using the top 2,000 most variable genes across 11 tissue types. A total of 3,983 samples were included in this analysis (see Materials and Methods for detailed sample counts per tissue). The first two components explained 37.6% (PC1) and 19.7% (PC2) of the variance, respectively. Samples from tissues containing epithelial secretory components, including breast, salivary gland, pancreas, stomach, small intestine, and skin, formed a coherent cluster that was separated from nonglandular tissues such as muscle, heart, esophagus, and adipose. Breast tissue samples occupied a position intermediate between adipose tissue and other epithelial secretory tissues, while remaining closer to the latter in PCA space. Breast, salivary gland, and pancreas samples showed relative proximity along the first two principal components (Fig. 1, Fig. S1).

**Fig. 2.**
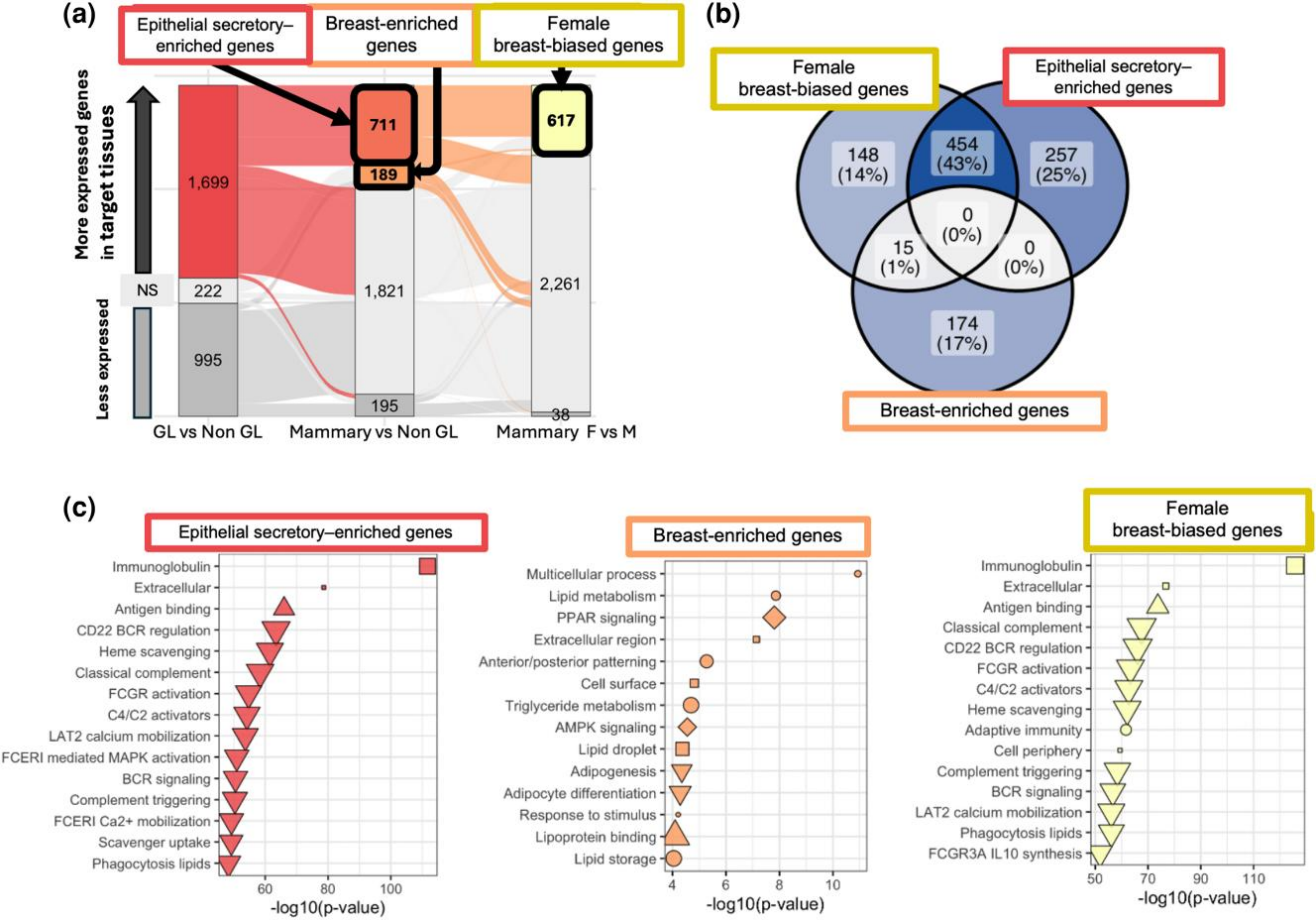
Differentially expressed gene categories across comparisons. a) Sankey diagram summarizing the number of differentially expressed genes (DEGs) identified in three pairwise comparisons: (i) tissues containing epithelial secretory components versus nonglandular tissues, (ii) breast tissue versus nonglandular tissues, and (iii) female versus male breast tissue samples. Bars indicate the number of upregulated (colored), downregulated (grey), and nonsignificant (NS; white) genes. Flows illustrate overlaps of gene categories across comparisons. b) Venn diagram illustrating the overlap among three categories of upregulated genes: epithelial secretory–enriched genes, breast-enriched genes, and female-biased genes. Numbers and percentages indicate the proportion of genes within each intersection. c) Gene Ontology (GO) and pathway enrichment analyses performed using g:Profiler for upregulated genes in the three DEG categories.

We next compared gene expression profiles across three expression categories defined by differential expression patterns across tissues and sex. Differentially expressed genes were grouped into three categories based on their patterns of relative enrichment. Genes upregulated in both breast tissue and other epithelial secretory tissues were classified as (1) “epithelial secretory–enriched genes” (711 genes). Genes showing higher expression in breast tissue relative to both nonglandular tissues and other epithelial secretory tissues were defined as (2) “breast-enriched genes” (189 genes). Finally, genes exhibiting higher expression in female relative to male breast samples were categorized as (3) “female breast-biased genes” (617 genes) (Fig. 2a; Table S1).

We observed substantial overlap between epithelial secretory–enriched genes and female breast-biased genes, whereas breast-enriched genes showed limited overlap with either category (Fig. 2b). Functional enrichment analysis further showed that these gene sets were associated with distinct functional annotations (Fig. 2c; Table S2). Epithelial secretory–enriched genes were predominantly linked to immune-related and extracellular functions, breast-enriched genes were associated with metabolic and endocrine pathways, and female breast–biased genes were strongly associated with immune- and complement-associated processes.

In the “epithelial secretory–enriched genes” group, we identified a cluster of genes associated with epithelial structure, secretion, and ion transport. This set included *DSP* (desmoplakin), *CDH1* (E-cadherin), *GRHL2*, and *BARX2*, which regulate epithelial integrity and branching morphogenesis, as well as several solute carriers *(SLC13A2, SLC5A1, SLC15A1*) and secretory enzymes (*PRSS8, PRSS22, SULT2B1*). In contrast, the “breast-enriched genes” group was characterized by genes associated with hormonal signaling, metabolic specialization, and epithelial differentiation. This set included hormone- and metabolite-responsive receptors such as *OXTR* (oxytocin receptor) and *HCAR1* (lactate receptor), together with the developmental regulator *TBX3* and the mammary-enriched long non-coding RNA *LINC00993*. Additional genes in this category included regulators of lipid metabolism, immune-associated processes, and cellular stress responses (e.g. *CREB3L4, STC2, CMTM7, SAA2,* and *SAA4*). The group further contained multiple transcription factors and structural or metabolic genes linked to epithelial organization and function, including *TWIST1, HOXD9, TRPS1, CLPSL1, PEMT,* and *SLC5A6*. Finally, the “female breast-biased genes” category was dominated by immunoglobulin genes, including IGHV, IGKJ, IGLJ, and IGKC family members, together with POU2AF1 and CD27.

### Single-cell Mapping and Bulk Deconvolution Provide a Cellular Context for Breast Tissue Gene Expression Patterns

Bulk RNA-seq data from GTEx breast tissue (*N* = 342) were deconvoluted using single-cell breast and adipose reference profiles from the CELLxGENE Discover portal to obtain descriptive estimates of cell-type composition across sex and age groups. Across all age groups and in both sexes, breast tissue samples contained a substantial adipose-associated fraction, accounting for approximately half of the inferred cellular composition (Fig. S2). While the relative proportion of adipose-associated cells varied across samples, the overall composition was similar across sex and age categories.

The remaining fraction consisted primarily of annotated epithelial and stromal-associated cell populations, including basal–myoepithelial and perivascular cells, with smaller contributions from macrophage and endothelial compartments. Given the age distribution of GTEx breast tissue donors, which is skewed toward middle-aged and older individuals, the inferred cellular composition is consistent with adult, nonlactational breast tissue samples. This compositional profile parallels the relative positioning of breast tissue samples between adipose and other epithelial secretory tissues in the global PCA (Fig. 1a).

**Fig. 3.**
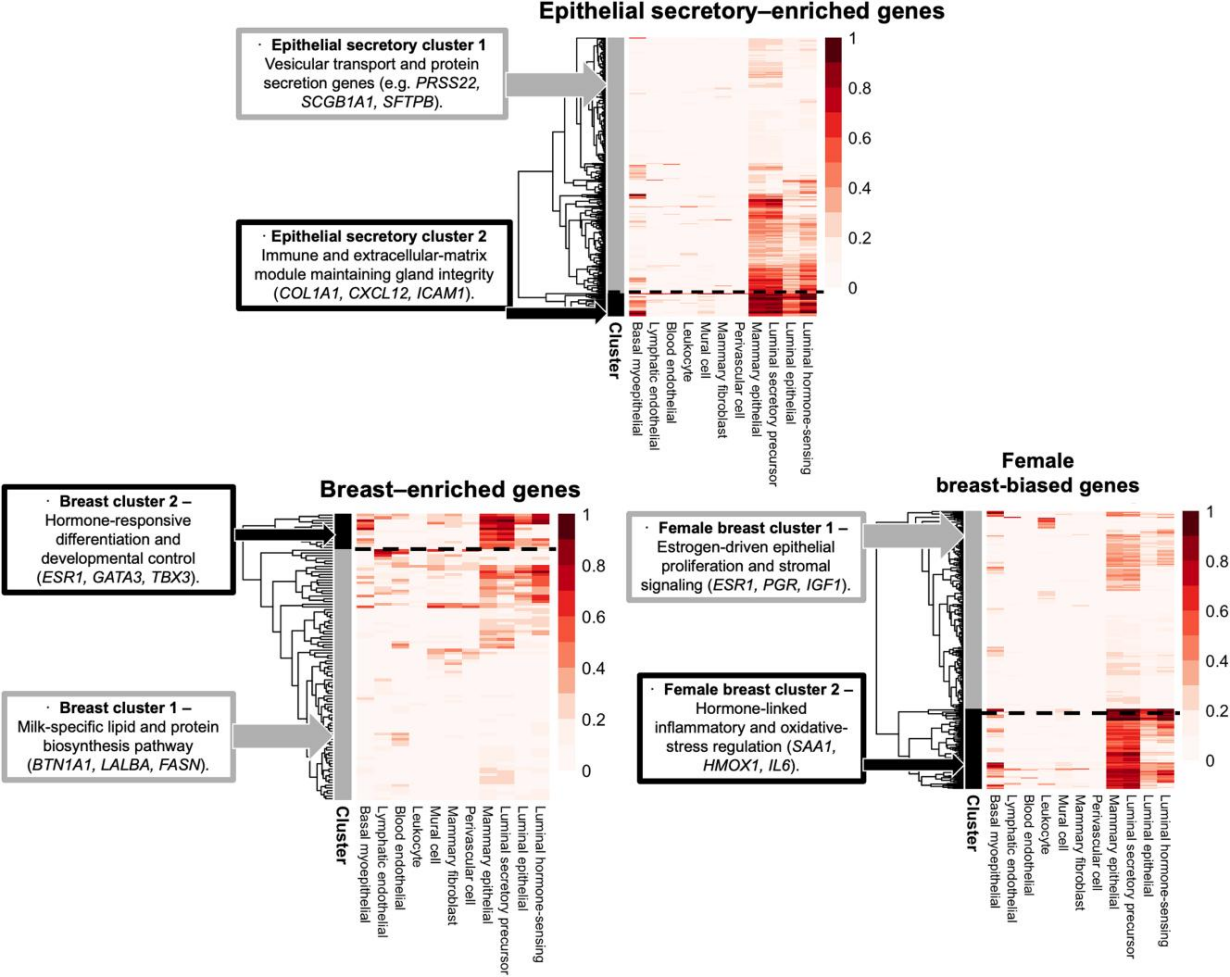
Single-cell expression patterns of upregulated gene sets. Heatmaps showing the proportion of single-cell populations expressing upregulated genes in each differentially expressed genes (DEGs) category: (i) genes upregulated across glandular tissues, (ii) genes upregulated only in mammary gland, and (iii) genes upregulated in female versus male mammary gland. Cell types include basal myoepithelial cells, luminal epithelial subtypes (hormone-sensing, secretory precursor, differentiated luminal), fibroblasts, endothelial cells (blood and lymphatic), mural cells, perivascular cells, and leukocytes. Expression levels are represented by scaled proportions of expressing cells (0–1), with rows corresponding to genes and columns to cell types.

To determine how each differential expression category is distributed across mammary cell populations, we examined the cellular expression patterns of genes belonging to the three defined groups: “epithelial secretory–enriched genes,” “breast-enriched genes,” and “female breast–biased genes.” For each gene, we calculated the detected fraction, defined as the proportion of cells within each annotated mammary cell type in which the gene was expressed (Fig. 3).

Within the “epithelial secretory–enriched genes” category, hierarchical clustering separated genes into two major clusters reflecting distinct single-cell detection patterns. One cluster comprised genes broadly detected across multiple mammary epithelial populations, including genes associated with vesicular transport and protein secretion, such as *PRSS22*. A second group showed higher detected fractions in annotated epithelial, secretory precursor, and hormone-sensing cell populations. These genes are associated with extracellular matrix and immune-related components, including *COL1A1*, *CXCL12*, and *ICAM1*.

For “female breast–biased genes,” cluster 1 showed a broad and heterogeneous expression pattern and included genes linked to mammary differentiation and metabolic specialization such as *BTN1A1* and *LALBA*. Cluster 2 genes were expressed in mammary epithelial and luminal secretory precursor cells and encompassed genes associated with hormone-responsive differentiation and developmental control, such as *ESR1, GATA3*, and *TBX3*.

Genes in the “female-biased genes” category also segregated into two major cellular expression patterns. Cluster 1 showed broad expression, and included estrogen-driven epithelial proliferation genes and stromal signaling genes, including *ESR1, PGR,* and *IGF1*. Cluster 2 genes were expressed in mammary epithelial and luminal secretory precursor cells and associated with hormone-linked inflammatory and oxidative stress regulation, including *SAA1, HMOX1,* and *IL6*.

### Comparative Genomics Analyses Compare Patterns of Evolutionary Conservation Across Expression-defined Breast Gene Categories

**Fig. 4.**
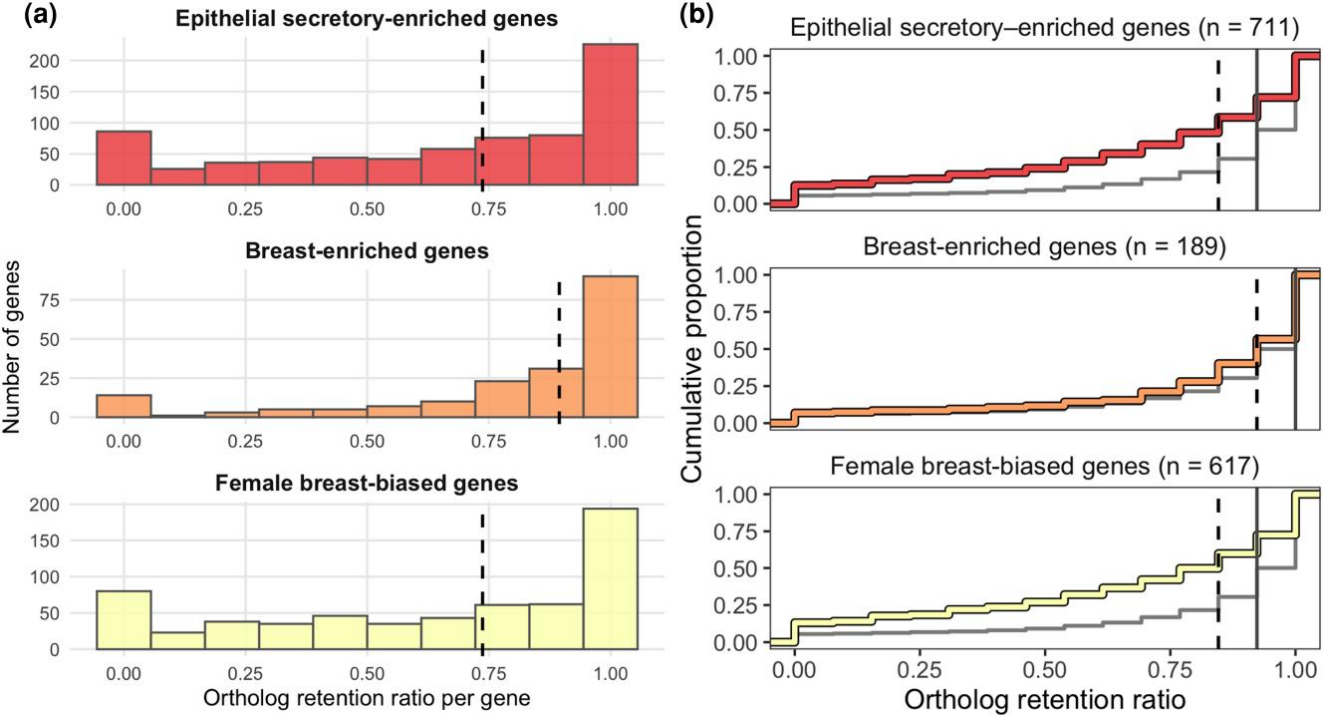
Evolutionary conservation of mammary gene categories across vertebrate species. For each gene category, the presence or absence of orthologs of human genes (genes in species that descend from a common ancestral gene following a speciation event) was examined across vertebrate species: *Mouse*, *Rat*, *Cattle*, *Goat*, *Sheep*, *Pig*, *Rabbit*, *Koala*, *Platypus*, *Chicken*, *Xenopus tropicalis*, *Zebrafish*, and *Medaka*. A gene's ortholog retention ratio represents the fraction of these species in which a corresponding ortholog is detected (0 = no orthologs retained, 1 = orthologs present in all species). a) Histograms display the distribution of retention ratios for three categories: epithelial secretory–enriched genes (red), breast-enriched genes (orange), and female breast-biased breast genes (yellow). Dashed vertical lines indicate the median retention ratio for each category. The *x* axis shows the ortholog retention ratio per gene, and the *y* axis indicates the number of genes in each bin. b**)** Distributional comparison of observed gene categories and size-matched random null sets. Empirical cumulative distribution functions show ortholog retention ratios for each observed gene category and its corresponding size-matched random null distribution. Colored lines indicate observed gene categories, and grey lines indicate random null distributions generated from 1,000 size-matched random gene sets. Dashed vertical lines indicate the median retention ratio of the observed category, and solid vertical lines indicate the median retention ratio of the corresponding random null distribution.

To examine patterns of evolutionary conservation among gene sets defined by tissue-associated expression, we compared the evolutionary conservation of the three gene categories identified in this study. Specifically, for genes classified as “epithelial secretory–enriched genes,” “breast-enriched genes,” and “female breast–biased genes,” we assessed the presence or absence of identifiable orthologs across a set of vertebrate species, including both mammalian and nonmammalian lineages spanning monotremes, birds, amphibians, and teleost fish (Fig. 4a and b, Table S3).

To compare these categories with genomic background, we generated 1,000 size-matched random gene sets for each category and compared the observed ortholog retention-ratio distribution with the corresponding random null distribution using Kolmogorov–Smirnov tests. These tests showed that epithelial secretory–enriched genes and female breast–biased genes differed significantly from their size-matched random null distributions (epithelial secretory–enriched: *D* = 0.279, *P* = 1.42 × 10^−48^; female breast–biased: *D* = 0.293, *P* = 2.91 × 10^−46^), whereas breast-enriched genes did not show a significant difference from the random null distribution (*D* = 0.0980, *P* = 0.0532) (Fig. 4b, Fig. S3).

A Kolmogorov–Smirnov test indicated a significant difference in the distribution of ortholog retention ratios between female breast–biased genes and breast-enriched genes (*D* = 0.217, *P* = 2.37 × 10^−6^), and between breast-enriched genes and epithelial secretory–enriched genes (*D* = 0.201, *P* = 1.21 × 10^−5^), whereas no significant difference was observed between female breast-biased genes and epithelial secretory–enriched genes (*D* = 0.0326, *P* = 0.874). Comparison of ortholog retention ratios indicated overall differences among the three gene categories (Kruskal–Wallis test, χ^2^ = 32.25, *df* = 2, *P* = 9.9 × 10^−8^). Consistent with this, pairwise comparisons showed that breast-enriched genes exhibited higher ortholog retention than both epithelial secretory–enriched genes and female breast–biased genes (Wilcoxon tests, *P* < 1 × 10^−6^), while no significant difference was detected between epithelial secretory–enriched genes and female breast–biased genes. This analysis was used to compare relative levels of evolutionary conservation across expression-defined gene sets, rather than to infer the evolutionary origin of specific tissues or traits.

### Mammary co-expression Modules and Their Overlap With Other Glandular Tissues

To examine patterns of gene co-expression in breast tissue and their similarity to those observed in other tissues, we performed weighted gene co-expression network analysis (WGCNA). WGCNA of breast tissue transcriptomes identified 14 co-expression modules, ranging in size from 232 to 2,429 genes (median ≈ 540 genes). The two largest modules (Modules 1 and 2) contained more than 1,000 genes each, whereas the remaining modules comprised between 200 and 900 genes.

**Fig. 5.**
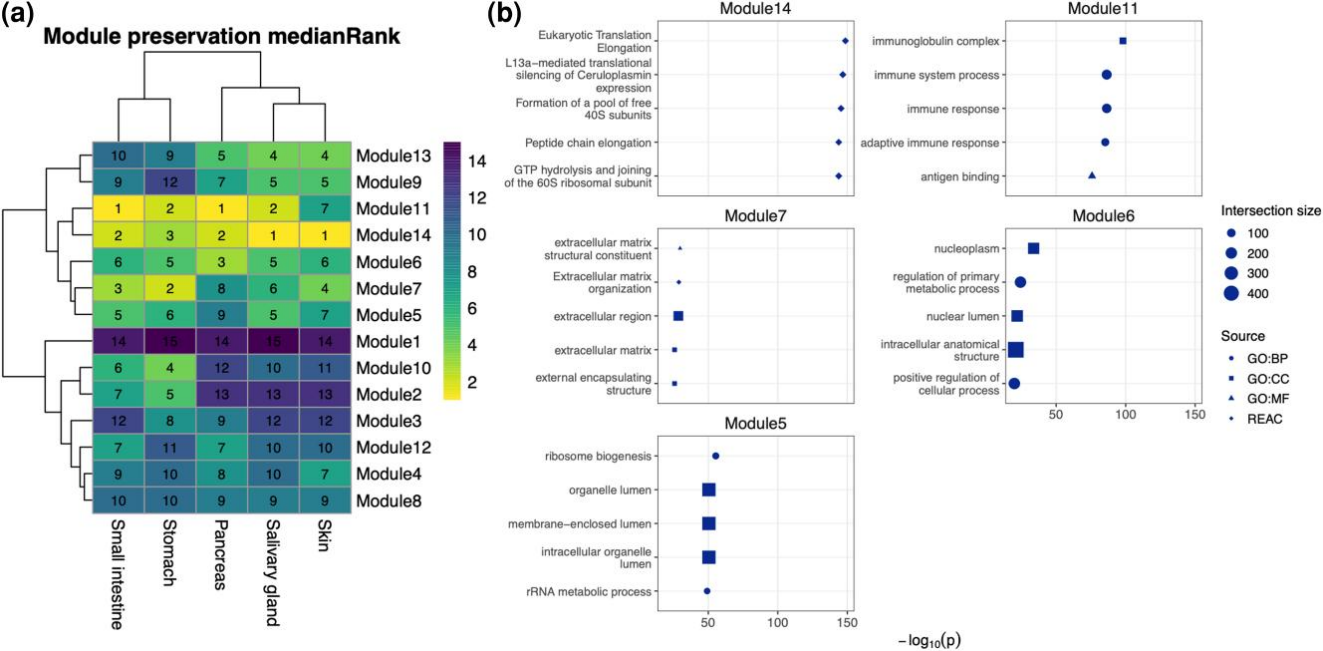
Cross-tissue comparison of mammary gene co-expression modules and their functional enrichment. a) Heat map showing relative module preservation based on medianRank for mammary gene co-expression modules across other tissues (skin, stomach, pancreas, salivary gland, and small intestine) calculated using the modulePreservation function in WGCNA with breast tissue as the reference network. Lower medianRank values indicate relatively stronger preservation of mammary co-expression network structure in the corresponding tissue. b) Functional enrichment analysis of selected mammary modules identified by medianRank analysis (Modules 14, 11, 7, 6, and 5). Enriched Gene Ontology (GO) and Reactome (REAC) terms are shown for each module. The *x* axis indicates −log10 (*P*-value) of enrichment, and point size represents the number of overlapping genes (intersection size). Different symbols denote annotation sources (GO:BP, GO:CC, GO:MF, REAC).

Module preservation analysis using breast tissue as the reference network revealed heterogeneous patterns of preservation across tissues. Several modules exhibited high preservation (Zsummary > 10) across multiple tissues, whereas others showed tissue-dependent variation (**Fig. S4**). In general, higher preservation values were observed between breast and skin and stomach, while lower preservation was observed between breast and salivary gland and intestine. Pancreas showed intermediate preservation. Because all examined modules showed strong preservation based on Zsummary, we further used medianRank to evaluate relative preservation among modules. Modules 14, 11, 7, 6, and 5 showed relatively low medianRank values across comparison tissues and were therefore selected for functional interpretation (Fig. 5a). The result of GO enrichment analysis for each module is shown in Fig. 5b. Module14 was enriched for translation-related processes, including “Eukaryotic Translation Elongation,” “peptide chain elongation,” and “formation of a pool of free 40S subunits.” Module 7 was associated with extracellular matrix–related terms, such as “collagen-containing extracellular matrix,” “extracellular matrix,” and “extracellular matrix organization.” Module 11 was enriched for immune-related processes, including “immune response,” “adaptive immune response,” “immunoglobulin complex,” and “antigen binding.” Module 11 showed higher preservation in intestine compared with other tissues. Module 6 was associated with intracellular and nuclear components, including “nucleoplasm,” “nuclear lumen,” and “intracellular anatomical structure,” as well as processes such as “regulation of primary metabolic process.” Module 5 was enriched for organelle- and ribosome-related terms, including “ribosome biogenesis,” “organelle lumen,” “membrane-enclosed lumen,” “intracellular organelle lumen,” and “rRNA metabolic process.”

To assess similarity of co-expression patterns across tissues, we calculated pairwise Jaccard indices between breast-derived modules and modules derived from 12 other GTEx tissues (Fig. S5). Overall overlap between breast modules and those from other tissues was low, with median Jaccard indices below 0.1 and no detectable difference between tissues containing epithelial secretory components and nonglandular tissues (Wilcoxon test, *P* > 0.05). Among the identified modules, breast Module 2 showed the highest and most recurrent overlap with modules from several tissues containing epithelial secretory components, including salivary gland, pancreas, stomach, and skin (Fig. S6a).

To describe the gene composition underlying these overlaps, we examined gene subsets shared between breast Module 2 and one or more comparison tissues. For each comparison tissue, only the module with the highest overlap with breast Module 2 was considered (Fig. S5b). Genes unique to the breast module were associated with pathways related to lipid metabolism and mitochondrial processes, including fatty acid biosynthesis, β-oxidation, and PPAR signaling, as well as hormone-associated signaling pathways. Gene sets shared between breast tissue and salivary gland or pancreas were associated with pathways related to vesicle trafficking, Golgi–ER processing, glycosylation, and exocytosis. Gene sets shared across breast tissue, salivary gland, and stomach were associated with extracellular matrix organization (Table S4).

## Discussion

Our analyses indicate that the breast tissue shares transcriptional features with other epithelial tissues containing secretory components in humans. Genes classified as epithelial secretory–enriched were expressed in the mammary gland as well as in salivary gland, pancreas, stomach, and skin, and were primarily associated with epithelial structure, secretory processes, extracellular matrix organization, and immune-related functions. This pattern indicates that a substantial fraction of breast tissue gene expression reflects transcriptional programs that are not unique to the mammary gland but are broadly deployed across epithelial secretory tissues. The epithelial secretory–enriched genes identified here are consistent with patterns reported in prior transcriptomic analyses in the mouse, which showed that exocrine organs such as the salivary gland, pancreas, and skin exhibit broadly similar epithelial gene expression profiles at the global level (Gluck et al. 2016). Single-cell reference mapping further indicated that many of these shared expression patterns are detectable in annotated epithelial cell populations, suggesting that common glandular features are largely carried by epithelial cell types with secretory or barrier-related functions.

Genes classified as glandular-enriched included regulators of epithelial adhesion and are associated with breast cancer, such as *CDH1* (Zhang et al. 2023; Dopeso et al. 2024), transcription factors implicated in epithelial differentiation including *GRHL2*  **(**Kumegawa et al. 2022; Wang et al. 2023) and *BARX2* (Sun et al. 2024). These genes have also been extensively studied in the context of breast cancer, where they are linked to epithelial identity and disease-associated transcriptional programs. Their enrichment across multiple epithelial secretory tissues suggests that such regulators form part of a shared epithelial gene expression core rather than representing mammary-specific innovations. Epithelial secretory–enriched genes also included solute carriers involved in epithelial transport, such as *SLC15A1*, which is well characterized for its duodenal-specific expression and role in peptide transport in the human upper gastrointestinal tract (Thwaites and Anderson 2007; Smith et al. 2013). The presence of such genes across secretory tissues is consistent with the reuse of established epithelial transport mechanisms in multiple physiological contexts.

In contrast, breast-enriched genes were enriched for regulators of hormonal responsiveness, metabolism, and epithelial differentiation. This group included hormone-related receptors such as *OXTR* (Reversi et al. 2005; Liu et al. 2020; Li et al. 2021, 2018) and *HCAR1*(Wagner et al. 2017; Jin et al. 2022; Mohammad Nezhady et al. 2023), transcription factors involved in mammary development and lineage specification including *TBX3* (Douglas and Papaioannou 2013), TRP*S1* (Cornelissen et al. 2020), and *TWIST1* (Foubert et al. 2010; Xu et al. 2017), and metabolic regulators such as *PEMT* (Resseguie et al. 2007) and *SLC5A6* (Montalbetti et al. 2014). The functional annotations of this gene set are consistent with known metabolic and endocrine features of breast tissue, particularly in relation to milk production (Uvnäs-Moberg and Eriksson 1996; Rezaei et al. 2016; McNamara and Huber 2018; Patel et al. 2019; Trott et al. 2022; Mohammad Nezhady et al. 2023; Jiang et al. 2025; Xiao et al. 2025). Several of these genes are known to respond to hormonal cues involved in mammary differentiation and secretory activation, and their expression patterns align with established roles of endocrine and metabolic regulation in mammary epithelial function (Li et al. 2018). These observations suggest that breast-enriched expression patterns are associated with metabolic and hormone-responsive processes layered onto a broader epithelial transcriptional background.

Genes exhibiting female breast-biased expression were dominated by B cell–related immunoglobulin genes, including *IGKC*, *IGHM*, *IGHV*, and *IGKJ* family members, which are well characterized as components of B cell– and antibody–mediated immune responses (Lycke et al. 2015; Hill et al. 2023). In addition, immune-associated regulators such as *POU2AF1* (Sun et al. 2024) and *CD27* (Jaeger-Ruckstuhl et al. 2024), which are primarily involved in T cell function and immune modulation, were also enriched. The observed female breast–biased expression of immune-related genes likely reflects differences in immune cell representation and activation states within breast tissue, in addition to sex-specific regulation of epithelial gene expression.

The observed co-expression module preservation patterns between breast and other epithelial secretory tissues were associated with functional categories. Module 14, enriched for translation-related processes, exhibited generally high preservation across tissues, particularly in skin and stomach. Module 7, associated with extracellular matrix organization, also showed relatively higher preservation in these tissues, indicating shared structural features. These patterns are consistent with the functional organization of the mammary gland as a secretory organ that integrates epithelial structure and extracellular support (Medina 1996; Patil et al. 2021).

Module 11, enriched for immune-related processes, displayed comparatively higher preservation in intestine and skin. The preservation of this immune-associated module is consistent with the role of the mammary gland in providing immunological protection through milk (Hassiotou and Geddes 2013). Module 5, enriched for ribosome biogenesis and organelle lumen–related processes, also showed relatively strong preservation, suggesting conservation of intracellular biosynthetic and ribonucleoprotein-associated functions across epithelial secretory tissues (Lakkaraju and Rodriguez-Boulan 2008).

Taken together, these results are consistent with an interpretation in which breast tissue gene expression reflects a combination of expression patterns shared with other epithelial secretory tissues and a more limited set of features showing relative enrichment in breast tissue. Rather than indicating a wholly distinct mammary-specific transcriptional program or widespread lineage-specific gene innovation, the breast transcriptome appears to be structured around conserved epithelial-associated gene sets with tissue-associated modulation linked to metabolic and hormonal functions. Consistent with these observations, hormone-associated regulatory mechanisms have been proposed as key drivers of tissue-specific switch-like expression patterns (Aqil et al. 2025), providing a plausible framework for interpreting mammary-specific and female breast–biased gene programs identified in the present study. This organization is compatible with the co-expression analyses presented in this study, which show partial overlap between breast-derived co-expression modules and those identified in other epithelial secretory tissues.

Within this framework, breast-associated genes may be interpreted as components of conserved gene sets that are differentially weighted or regulated in breast tissue. One example is *STC2 (Stanniocalcin 2),* a mammary-specific gene in our analysis. Although stanniocalcins originated as calcium-regulatory hormones in nonmammalian vertebrates, STC2 is expressed in adult, nonlactational mammary tissue, and its expression pattern is consistent with the reuse of an ancestral endocrine regulator in a breast-associated regulatory context (Hasilo et al. 2005).

From an evolutionary perspective, the patterns observed here are compatible with models in which tissue specialization arises through regulatory modification of conserved gene repertoires. Studies in both plants and animals have shown that novel organ morphologies often emerge through changes in the deployment, connectivity, or regulation of preexisting gene modules rather than through the invention of new genes (e.g. “old genes, new functions”) (Rosin and Kramer 2009; Pires and Dolan 2012; Defoort et al. 2018; Uebbing et al. 2024). In this context, breast tissue can be viewed as assembling transcriptional features drawn from broadly conserved epithelial and secretory gene programs, with specialization arising through regulatory modifications rather than lineage-specific gene invention.

However, it is important to note that our analysis of ortholog presence reflects the evolutionary conservation of gene repertoires and does not directly assess the conservation of regulatory interactions or module structure across deep evolutionary timescales. Future comparative work integrating multi-tissue transcriptomic datasets across a broad range of mammalian and nonmammalian species and individuals would help determine the extent to which regulatory architectures associated with mammary and breast tissue are conserved or have been independently assembled from shared gene repertoires.

Together, these findings situate breast tissue gene expression within a continuum of epithelial tissues containing secretory components, highlighting shared transcriptional features alongside tissue-associated expression patterns. Viewing breast gene expression through this comparative framework provides a basis for integrating transcriptomic observations across tissues, and may be applicable to the study of other epithelial organs that exhibit both shared molecular features and tissue-specific physiological specialization. Viewing breast gene expression through this comparative and evolutionary framework provides a basis for interpreting mammary gland identity as an outcome of regulatory modularity, while leaving the evolutionary origin of the regulatory architectures as an open question. This study suggests that similar approaches may be informative for understanding the evolution of other lineage-specific organs that combine conserved molecular architectures with tissue-specific physiological functions.

## Materials and Methods

### Dataset Selection and Tissue Group Definition

Bulk RNA-seq expression data were obtained from the GTEx v10 dataset. Tissues were categorized into two analysis groups for comparative purposes, referred to here as tissues containing epithelial secretory components and nonglandular tissues, for differential expression analyses.

The epithelial secretory tissue group comprised six tissues characterized by epithelial origin and secretory function: breast—mammary tissue; minor salivary gland; skin—not sun exposed (suprapubic); and pancreas, stomach, and small intestine—terminal ileum. Breast, salivary gland, and skin were included due to their abundant exocrine acinar or ductal structures (Pia-Foschini et al. 2003; Wang et al. 2017), despite differences in secreted products. Pancreas, stomach, and small intestine represent classical exocrine organs involved in digestive enzyme and fluid secretion. GTEx breast samples are derived from adult donors, and pregnancy or lactation status is not publicly available. Accordingly, breast samples are treated as representing adult, nonlactational breast tissue.

The nonglandular group consisted of six tissues used as comparative reference tissues lacking prominent epithelial secretory structures: muscle—skeletal, heart—left ventricle, artery—tibial, esophagus—muscularis, adipose—subcutaneous, and adipose—visceral (omentum).

### Data Filtering

Samples were filtered using GTEx-provided quality metrics. Inclusion required RNA integrity number (SMRIN ≥ 6.0), mapping rate (SMMAPRT ≥ 0.8), sequencing efficiency (SMEXPEFF ≥ 0.6), at least 50 million total reads (SMRDTTL), and 50 million uniquely mapped nonduplicate reads (SMMPPDUN). Samples were required to express at least 10,000 genes (SMGNSDTC).

Samples with high duplicate rates (SMDPMPRT > 0.6), elevated base mismatch rates (SMBSMMRT > 0.01), excessive chimeric reads (SMCHMRT > 0.015), UniVec contamination (SMUVCRT > 0.1), or extreme 3′ bias (SM3PBMN outside 0.4–0.75) were excluded. A minimum estimated library complexity of 30 million unique fragments (SMESTLBS) was required. Samples missing key metadata required for modeling were excluded. After filtering, samples from 12 tissue types were retained.

Finally, a total of 3,983 samples, comprising 966 adipose tissue, 537 blood vessel, 342 breast, 393 esophagus, 166 heart, 544 muscle, 76 pancreas, 142 salivary gland, 486 skin, 168 small intestine, and 163 stomach samples.

### Covariates and Sample Metadata

Sample-level biological covariates included sex, age category, and Hardy death classification. The variable *SEX* is coded as 1 for male and 2 for female, while *AGE* corresponds to decade intervals (e.g. 20–29, 30–39, etc.). The *DTHHRDY* score describes the mode of death on a five-level Hardy scale, which serves as a proxy for postmortem condition.

Technical covariates included collection center (SMCENTER), nucleic acid batch (SMNABTCH), gene expression batch (SMGEBTCH), analyte type (ANALYTE_TYPE), and storage condition (SMAFRZE). Sequencing and RNA quality metrics used either for filtering or modeling included SMRIN, SMTSISCH, SMTSPAX, SMRDTTL, SMMAPRT, SMMPPDUN, SMEXPEFF, SMEXNCRT, SMRRNART, SMCHMRT, and SM3PBMN.

For differential expression modeling, SEX, AGE, SMCENTER, and SMEXPEFF were included as fixed effects. Samples missing any of these covariates were excluded prior to normalization and statistical analysis.

### Differential Expression Analysis

Raw RNA-seq read count data from thirteen human tissues were obtained from the GTEx v10 dataset. Count matrices were imported directly from the compressed GTEx.gct.gz files, with sample columns matched to the curated metadata. The combined matrix was then used to create *DGEList* objects in *edgeR* (Robinson et al. 2010), and lowly expressed genes were excluded using the filterByExpr() function with the relevant grouping factor. Counts were normalized by the trimmed mean of M-values (TMM) method to correct for differences in library size and sequencing depth.

Differential expression analyses were performed under several contrasts. In the first analysis, tissues containing epithelial secretory components were compared to nonglandular tissues to identify genes showing elevated expression across this tissue set. In the second analysis, breast tissue samples were contrasted against nonglandular tissues to identify genes showing relative enrichment in breast tissue within this comparison framework. A third comparison was carried out within breast tissue samples to detect sex-associated expression differences between male and female samples. For each analysis, a linear modeling framework was applied using the limma–voom pipeline. Coefficients corresponding to the main factor of interest were extracted from the fitted model. Genes were considered significantly differentially expressed when the absolute log_2_ fold change exceeded one and the false discovery rate (Benjamini–Hochberg adjusted *P*-value) was below 0.001.

Differentially expressed genes were grouped into three expression categories defined operationally by their patterns across these contrasts. Genes upregulated in both breast tissue and other epithelial secretory tissues were classified as (1) “epithelial secretory–enriched genes.” Genes upregulated in breast tissue relative to both nonglandular tissues and other epithelial secretory tissues were defined as (2) “breast-enriched genes.” Finally, genes exhibiting higher expression in female relative to male breast samples were categorized as (3) “female breast–biased genes.”

### Integration of Bulk RNA-Seq Data and Single-cell RNA-Seq Data

Single-cell RNA-seq datasets of breast tissue and subcutaneous adipose tissue (Reed et al. 2024; Lazarescu et al. 2025) were downloaded at CELLxGENE Discover portal (CZI Cell Science Program et al. 2025) as reference expression resources to contextualize bulk RNA-seq data. We used publicly available single-cell RNA-seq data from the Human Breast Cell Atlas (HBCA, global dataset, ∼803,000 cells), generated from normal adult human breast tissue and snRNA-seq of human subcutaneous adipose tissue—all cells (37,879 cells, GSE281356) (Reed et al. 2024; Lazarescu et al. 2025). In this study, we used subcutaneous adipose tissue as the comparator for reference breast adipose data because the adipose component of the human breast is anatomically and histologically classified as subcutaneous adipose tissue. The human breast is composed of glandular (mammary) and adipose tissue embedded in a stromal framework beneath the skin, with adipose tissue forming the major volume of the breast outside the glandular component (Nickell and Skelton 2005). Adipose tissue located beneath the skin is defined as subcutaneous adipose tissue, in contrast to visceral adipose tissue, which is localized around internal organs within the abdominal cavity (Ibrahim 2010).

Cell-type composition of bulk mammary gland RNA-seq data was estimated using a reference-based deconvolution approach implemented in the DeconRNA-seq (Gong and Szustakowski 2013) package (Bioconductor). Reference expression matrices were derived from single-cell RNA-seq datasets of human mammary and subcutaneous adipose tissues (Reed et al. 2024; Lazarescu et al. 2025). For each dataset, mean expression profiles were calculated for every annotated cell type, with genes as rows and cell types as columns.

From the resulting deconvolution output, adipocyte-related fractions were obtained by summing columns annotated as “adipocyte,” “mesenchymal stem cell of adipose tissue,” or similar lipid-associated terms. These estimated fractions were then integrated with GTEx sample metadata containing sex and age information. Age was converted to numeric values where possible and categorized into five intervals (≤30, 31–40, 41–50, 51–60, and ≥61 years). While pregnancy/lactation status was not available in the sample metadata, among female donors, samples from individuals aged 39 years or younger accounted for approximately 17% of the dataset, with the majority derived from donors aged 40 years and above.

### Single-cell Expression Profiling and Gene Clustering

Single-cell reference data were processed in R using the *zellkonverter* (https://bioconductor.org/packages/zellkonverter) and *DelayedArray* (https://code.bioconductor.org/browse/DelayedArray/) packages to enable efficient handling of large HDF5-backed matrices. Annotated expression data were imported from the.h5ad file, and cells were grouped according to their annotated cell type. To focus on biologically relevant features, only genes previously identified as differentially expressed were retained. The investigation of single-cell data was restricted to genes identified in bulk differential expression analyses, in order to examine how bulk-defined gene sets map onto reference cell populations.

For each annotated cell type, we calculated the detected fraction, defined as the proportion of cells in which a given gene was expressed (nonzero counts). This metric was used as comparative measure of gene activity across cell populations. Detected-fraction matrices were subjected to hierarchical clustering (Euclidean distance, complete linkage) to group genes with similar patterns across annotated cell types. Clustering was used to summarize broad patterns of cell-type association. Each dendrogram was divided into three clusters, and functional enrichment analyses were performed for each cluster using *g:Profiler* (*gprofiler2* package (Kolberg et al. 2020)) with Gene Ontology (BP, MF, CC), KEGG, and Reactome databases. Enrichment significance was assessed using the false discovery rate (FDR) correction.

### Evolutionary Conservation Analysis

To assess patterns of evolutionary conservation among gene sets defined by breast-associated differential expression, we compiled a list of human genes differentially expressed in breast tissue and other epithelial secretory tissues and examined their orthologous relationships across representative vertebrate lineages using Ensembl BioMart (release ≥ 111). The ortholog presence matrix included 13 species covering major vertebrate clades: Eutheria (*mouse, Mus musculus; rat, Rattus norvegicus; rabbit, Oryctolagus cuniculus; pig, Sus scrofa; cattle, Bos taurus; goat, Capra hircus; sheep, Ovis aries*), Marsupialia (koala, *Phascolarctos cinereus*), Monotremata (platypus, *Ornithorhynchus anatinus*), Aves (chicken, *Gallus gallus*), Amphibia (*Xenopus tropicalis*), and Teleostei (zebrafish, *Danio rerio*; medaka, *Oryzias latipes*).

For each human Ensembl gene ID, ortholog presence (1) or absence (0) was determined in each species, resulting in a binary matrix. A total of 11,125 human genes with detectable expression and included in the differential expression analysis were used as the background gene set. For each gene, an ortholog retention ratio was calculated as the proportion of surveyed species in which an identifiable ortholog was present. Genes were grouped according to their expression-based categories, including epithelial secretory–enriched genes, breast-enriched genes, and female breast–biased genes, defined on the basis of differential expression across glandular, mammary, and sex-based contrasts. Median ortholog retention ratios were calculated for each gene set. This analysis was used to compare relative levels of evolutionary conservation across expression-defined gene sets, rather than to infer the evolutionary origin of specific tissues or traits.

To evaluate whether the observed retention patterns differed from random expectation, size-matched random gene sets (*n* = 711 for epithelial secretory–enriched genes, *n* = 617 for female breast–biased genes, and *n* = 189 for breast-enriched genes) were generated by sampling from this background set of 11,125 expressed genes 1,000 times for each category. For each iteration, the median ortholog retention ratio was calculated, and the observed values were compared with the resulting null distributions. Differences in ortholog retention among categories were assessed using a Kruskal–Wallis test, followed by pairwise Wilcoxon rank–sum tests. In addition, pairwise Kolmogorov–Smirnov tests were performed to compare the full distributions of ortholog retention ratios between categories. In addition, Kolmogorov–Smirnov tests were performed between observed and random retention ratio distributions for each category.

### Weighted Gene co-expression Network Analysis (WGCNA) and Cross-tissue Module Comparison

Normalized gene expression counts from the GTEx dataset were used to construct tissue-specific co-expression networks using the WGCNA package (https://cran.r-project.org/web/packages/WGCNA/index.html) (v1.72). For the breast tissue, cell-type composition effects estimated by DeconRNA-seq were regressed out to reduce variability associated with endothelial and macrophage fractions, using removeBatchEffect in the limma package. using removeBatchEffect in the limma package (Ritchie et al. 2015). Lowly expressed genes were first filtered using the filterByExpr() function in edgeR. After this, log-transformed CPM values of each gene were filtered to retain genes expressed in at least 20% of samples. This threshold was chosen to avoid excluding genes with subgroup-restricted expression patterns arising from unobserved biological conditions, since the phenotype information available in the GTEx in the metadata is limited. A signed network was built with bicor correlation and dynamic tree cutting (minimum module size = 120, merge cut height = 0.35). Modules were labeled in descending order of size (Module 1, Module 2, …), and module eigengenes (MEs) were extracted for downstream analyses.

To evaluate similarity of co-expression structure across tissues, module assignments from the mammary gland were compared with those from other GTEx tissues. For each tissue, Jaccard similarity was computed between all pairs of mammary and tissue modules based on shared gene memberships. For each breast module, the maximum Jaccard index across modules in a given tissue was retained as a summary measure of overlap (“best-per-tissue” score). Permutation tests (*n* = 2,000) were performed by shuffling gene labels in the comparison tissue while preserving module sizes, yielding empirical *P*-values and FDR-corrected significance estimates.

Module preservation analysis was performed to assess conservation of co-expression network structure across tissues. Using breast tissue as the reference network, module assignments defined in the mammary gland were evaluated in other tissues using the modulePreservation function in WGCNA(Langfelder and Horvath 2008). Expression matrices were restricted to genes shared across all tissues to ensure comparability. Preservation statistics were calculated using a signed network with bicor correlation, and significance was assessed based on Zsummary statistics derived from permutation testing (*n* = 200). Zsummary values greater than 10 were interpreted as strong preservation, values between 2 and 10 as moderate preservation, and values below 2 as weak or no preservation (Langfelder et al. 2011). Because most modules showed strong preservation according to Zsummary, relative preservation among modules was additionally evaluated using medianRank statistics, where lower values indicate stronger relative preservation independent of module size.

### Use of Large Language Model

We used ChatGPT (version 5.2, OpenAI) to assist with code refactoring, language editing, and proofreading. All scientific interpretations, analyses, and conclusions are the responsibility of the authors.

## Supplementary Material

evag130_Supplementary_Data

## Data Availability

All data and analysis scripts supporting this study are available at: https://github.com/mariesaitou/paper_2025-/tree/main/breast_comparative. The study utilized publicly accessible human transcriptomic datasets from the GTEx Consortium (v10) and the CZ CELLxGENE Discover portal, including the Human Breast Cell Atlas and human subcutaneous adipose tissue datasets (See Materials and Methods). No additional restrictions apply to data sharing.
