# Supplementary material for: Comparative Transcriptomics Suggests that Breast Secretory Epithelium Reuses Gene Repertoires Conserved Across Vertebrates Beyond Mammals
Source: Genome Biol Evol. 2026 May 29;18(6):evag130. doi: 10.1093/gbe/evag130 (PMC13271094; doi:10.1093/gbe/evag130)
Supplement: evag130_Supplementary_Data [file evag130_supplementary_data.zip › mammary_SFigures_R2_rev.docx]

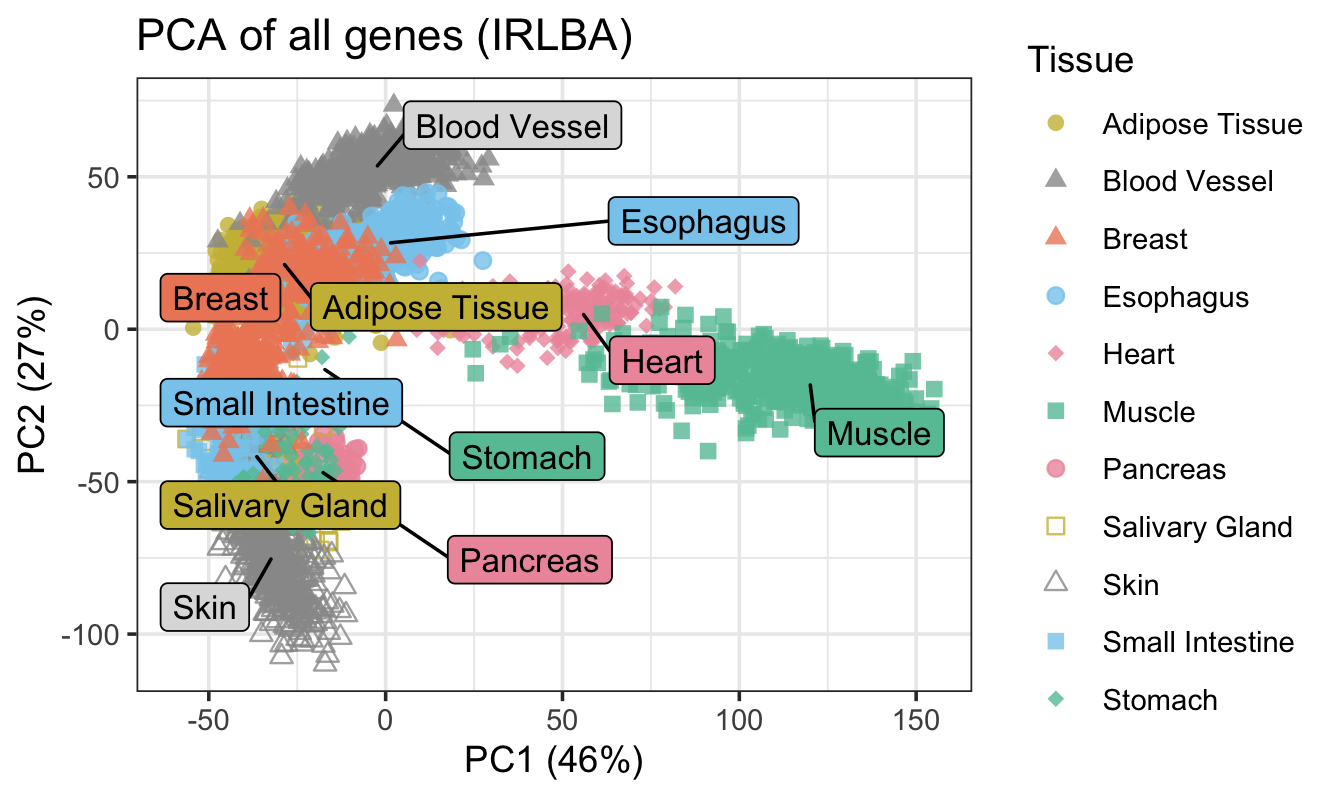


**Figure S1. Principal component analysis (PCA) of tissue transcriptomes using all expressed genes**
Principal component analysis (PCA) was performed using all expressed genes across tissues, to summarize global patterns of transcriptome variation across samples. Each point represents a tissue sample, and tissues are colored according to their anatomical source. Tissues containing epithelial secretory components, including breast, salivary gland, pancreas, stomach, small intestine, and skin, cluster separately from non-glandular tissues, including muscle, heart, blood vessel, esophagus, and adipose. The x-axis (PC1) and y-axis (PC2) show the first and second principal components, which explain 46% and 27% of the total expression variance, respectively. Samples that are closer together in this plot have more similar global expression profiles, whereas those farther apart differ more strongly in their transcriptome composition. Colored clusters correspond to distinct tissue types. Labels indicate the centroids of major tissue groups.


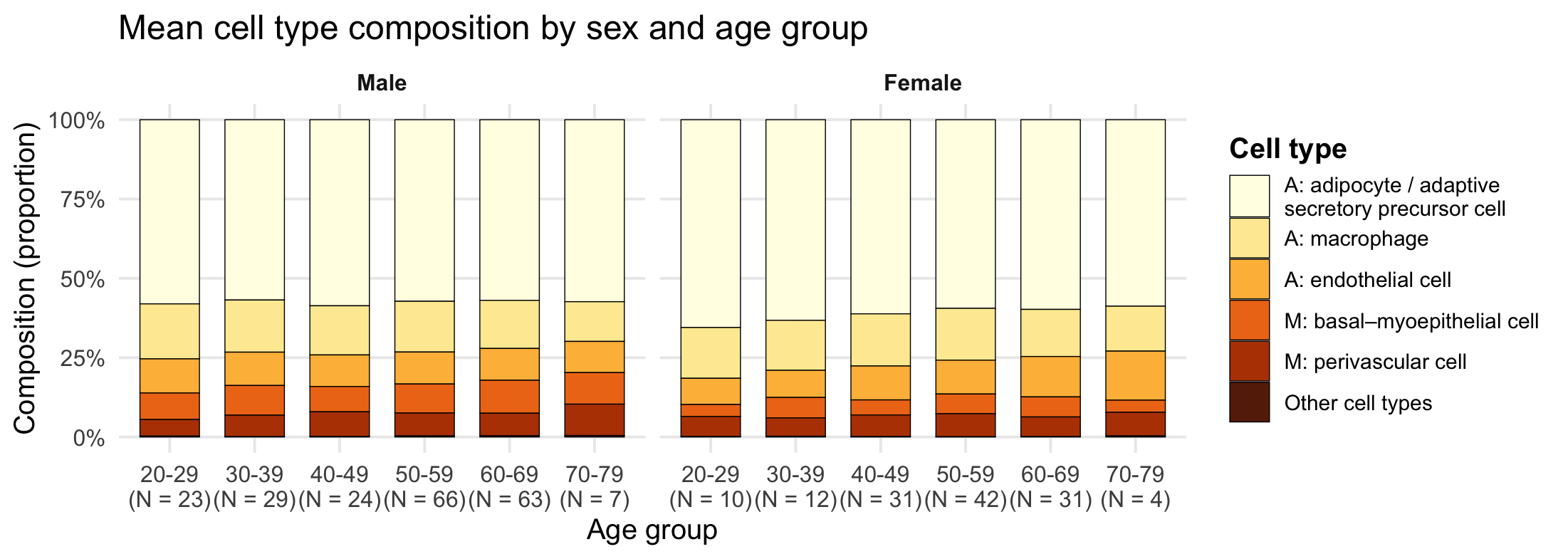


**Figure S2. Mean cell type composition in breast tissue by sex and age group**

Bulk RNA-seq data from GTEx breast tissue (N=342) were deconvoluted using single-cell reference expression profiles to estimate relative cell-type composition. Stacked bar plots show mean fractional compositions averaged within each sex (male, female) and age group (10-year bins), with the y-axis normalized to 100%. Colors denote inferred cell populations: adipocyte / adaptive secretory precursor cell (A: Adipose), macrophages (A: Adipose), endothelial cells (A: Adipose), basal–myoepithelial cells (M: Mammary),perivascular cells (M: Mammary), and minor fractions classified as “Other cell types.” Single-cell RNA-seq reference datasets of  breast tissue and subcutaneous adipose were downloaded at CELLxGENE Discover portal (CZI Cell Science Program et al. 2025).


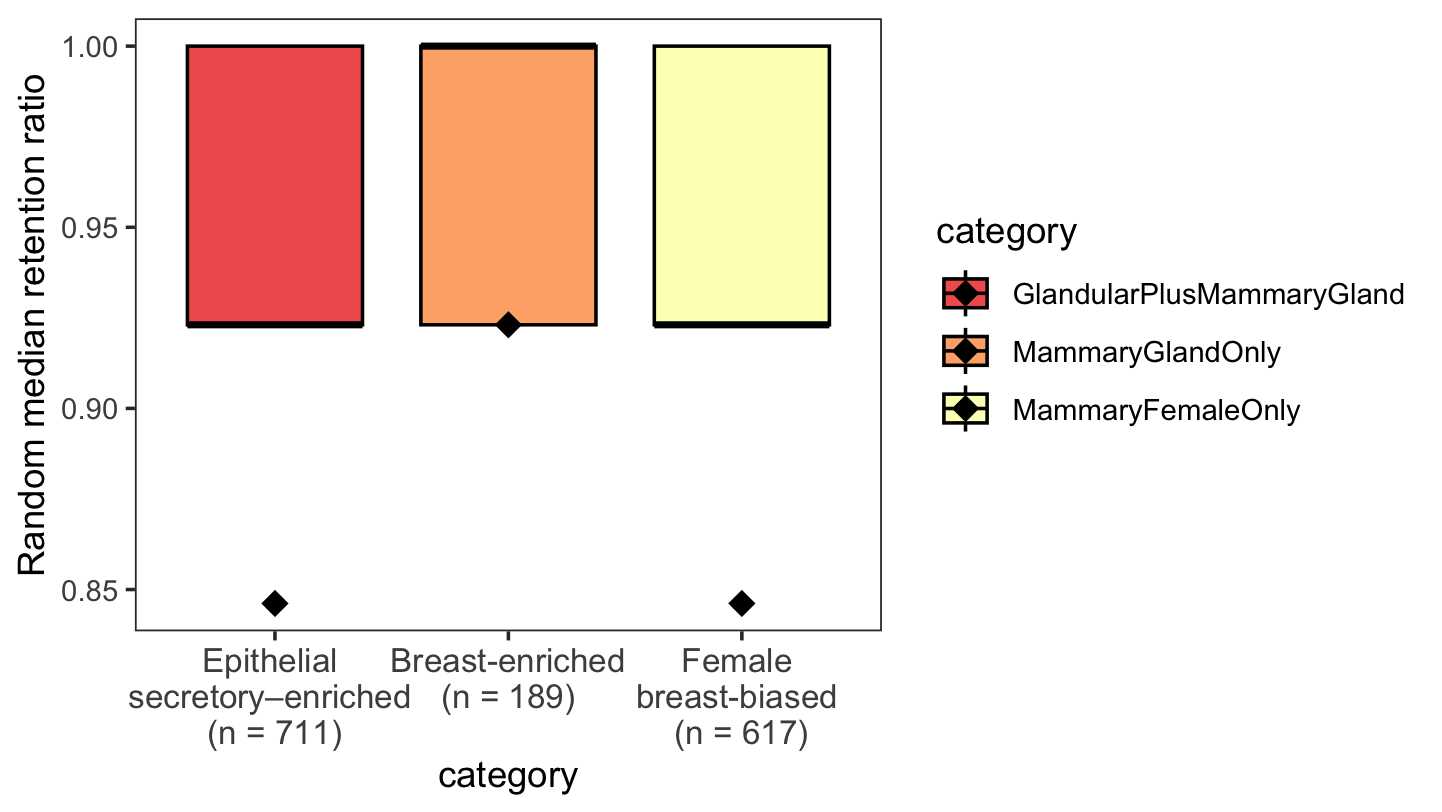


**Figure S3.** Boxplots show the distribution of median ortholog retention ratios from 1,000 random gene sets matched in size to each category. Black diamonds indicate the observed median retention ratio for each gene category.


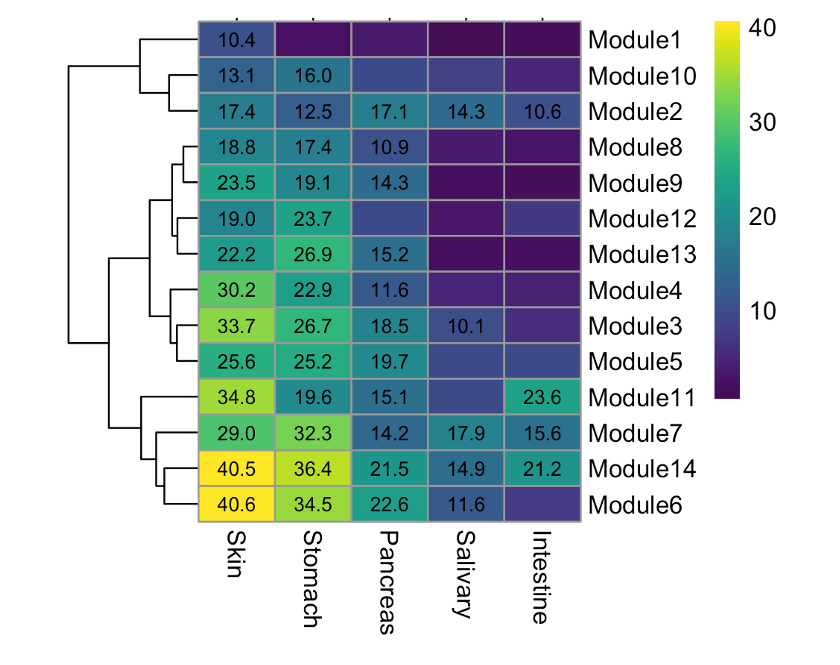


**Figure S4.** Heatmap showing module preservation statistics (Zsummary) for mammary gene co-expression modules across other tissues (skin, stomach, pancreas, salivary gland, and small intestine), calculated using the modulePreservation function in WGCNA with breast tissue as the reference network. Higher Zsummary values indicate stronger preservation of mammary co-expression network structure in the corresponding tissue.

**Figure S5. Cross-tissue overlap of breast co-expression modules.**

Heatmap showing Jaccard indices between breast tissue co-expression modules and modules derived from other GTEx tissues. For each comparison tissue, the Jaccard index represents the maximum overlap observed between a given breast module and any module from that tissue (“best-per-tissue” overlap). Rows correspond to comparison tissues, and columns correspond to breast co-expression modules. Tissues are annotated as tissues containing epithelial secretory components or non-glandular tissues based on the tissue grouping used in this study. Warmer colors indicate higher Jaccard similarity.

CZI Cell Science Program et al. 2025. CZ CELLxGENE Discover: a single-cell data platform for scalable exploration, analysis and modeling of aggregated data. Nucleic Acids Res. 53:D886–D900.


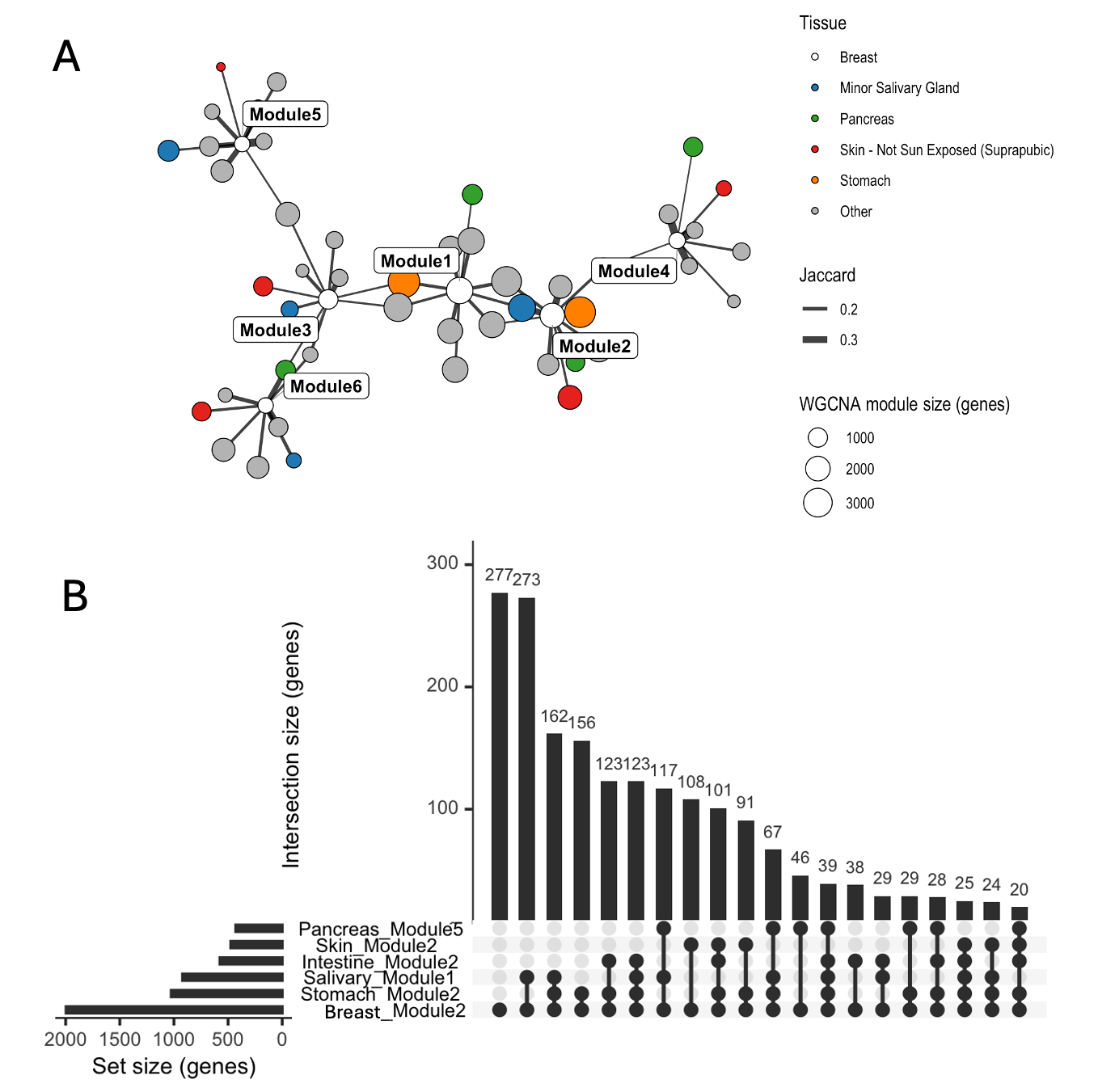


**Figure S6. A.** Network visualization of breast-specific gene co-expression modules (Modules 1–6) identified using WGCNA and their best-matching modules across other human tissues. Each node represents a gene co-expression module, defined as a cluster of genes that show highly correlated expression patterns within a given tissue. Node size corresponds to WGCNA module size (number of genes). Node color indicates the tissue of origin, with white nodes representing breast modules and colored nodes representing modules from other glandular tissues. Edge thickness is proportional to the Jaccard index value, such that thicker lines indicate stronger gene overlap between modules.

**B.** UpSet plot summarizing gene set intersections between breast modules and their top-matching modules from other tissues. Each filled dot in the bottom matrix represents the inclusion of a particular module in a given intersection set, and connecting lines indicate combinations of modules that share overlapping genes. The vertical bars above the matrix show the number of genes (intersection size) for each specific module combination, while the horizontal bars on the left indicate the total number of genes (set size) contained in each module.
